# Functional compared to anatomical imaging in the initial evaluation of patients with suspected coronary artery disease: An international, multi-center, randomized controlled trial (IAEA-SPECT/CTA study)

**DOI:** 10.1007/s12350-016-0664-3

**Published:** 2016-10-28

**Authors:** Ganesan Karthikeyan, Barbara Guzic Salobir, Borut Jug, Niveditha Devasenapathy, Erick Alexanderson, Joao Vitola, Otakar Kraft, Elgin Ozkan, Saket Sharma, Gaurav Purohit, Maja Dolenc Novak, Aloha Meave, Sergio Trevethan, Rodrigo Cerci, Sandra Zier, Lucia Gotthardtová, Tomáš Jonszta, Timucin Altin, Cigdem Soydal, Chetan Patel, Gurpreet Gulati, Diana Paez, Maurizio Dondi, Ravi Kashyap

**Affiliations:** 1 Department of Cardiology, All India Institute of Medical Sciences, New Delhi, 110029 India; 2 Department of Nuclear Medicine, All India Institute of Medical Sciences, New Delhi, India; 3 Department of Cardiac Radiology, All India Institute of Medical Sciences, New Delhi, India; 4 Department of Nuclear Medicine, University Medical Centre Ljubljana, Ljubljana, Slovenia; 5 Department of Vascular Medicine, University Medical Centre Ljubljana, Ljubljana, Slovenia; 6 Indian Institute of Public Health-Delhi, Gurgaon, India; 7 Department of Nuclear Medicine, Ignacio Chávez National Institute of Cardiology, Mexico City, Mexico; 8 Department of Radiology, Ignacio Chávez National Institute of Cardiology, Mexico City, Mexico; 9 Quanta Diagnóstico & Terapia, Curitiba, Brazil; 10 Department of Nuclear Medicine, Faculty Hospital Ostrava, Ostrava, Czech Republic; 11 Department of Cardiology, Faculty Hospital Ostrava, Ostrava, Czech Republic; 12 Department of Radiology, Faculty Hospital Ostrava, Ostrava, Czech Republic; 13 Department of Nuclear Medicine, Ankara University Medical Faculty, Ankara, Turkey; 14 Department of Cardiology, Ankara University Medical Faculty, Ankara, Turkey; 15 Division of Human Health, Department of Nuclear Sciences and Applications, International Atomic Energy Agency, Vienna, Austria

**Keywords:** Myocardial perfusion imaging—SPECT, computed tomography, coronary artery disease

## Abstract

**Objective:**

To test the hypothesis that, in the initial evaluation of patients with suspected coronary artery disease (CAD), stress myocardial perfusion imaging (MPI) would result in less downstream testing than coronary computed tomographic angiography (CCTA).

**Methods:**

In this international, randomized trial, mildly symptomatic patients with an intermediate likelihood of having CAD, and asymptomatic patients at intermediate risk of cardiac events, underwent either initial stress-rest MPI or CCTA. The primary outcome was downstream noninvasive or invasive testing at 6 months. Secondary outcomes included cumulative effective radiation dose (ERD) and costs at 12 months.

**Results:**

We recruited 303 patients (151 MPI and 152 CTA) from 6 centers in 6 countries. The initial MPI was abnormal in 29% (41/143) and CCTA in 56% (79/141) of patients. Fewer patients undergoing initial stress-rest MPI had further downstream testing at 6 months (adjusted OR 0.51, 95% CI 0.28-0.91, *P* = 0.023). There was a small increase in the median cumulative ERD with MPI (9.6 vs. 8.8 mSv, *P* = 0.04), but no difference in costs between the two strategies at 12 months.

**Conclusion:**

In the management of patients with suspected CAD, a strategy of initial stress MPI is substantially less likely to require further downstream testing than initial testing with CCTA. Trial registration: clinicaltrials.gov identification number NCT01368770.

**Electronic supplementary material:**

The online version of this article (doi:10.1007/s12350-016-0664-3) contains supplementary material, which is available to authorized users.

## Introduction

Functional testing by stress myocardial perfusion imaging (MPI) and anatomical imaging by coronary computed tomography angiography (CCTA) are often used interchangeably in the initial evaluation of patients suspected to have coronary artery disease (CAD). In patients with an intermediate likelihood of having CAD, the results of functional testing provide important diagnostic and prognostic information. This information is usually sufficient to determine the need for further invasive testing and revascularization.^1^ Coronary CTA provides accurate anatomical information regarding the extent and severity of CAD.^2^,^3^ But this often needs to be supplemented by the documentation of typical symptoms, or the objective demonstration of ischemia by further testing, before management decisions can be made. Moreover, the mere identification of anatomical stenosis may often lead to revascularization without the assessment of its functional significance.^4^ Therefore, a strategy of initial evaluation by CCTA may result in greater downstream testing and revascularization, resulting in increased healthcare costs.^3^,^5^ On the other hand, CCTA may also detect the presence of hemodynamically insignificant coronary lesions which may be prognostically important,^6^ and although unproven, may potentially benefit from intensive medical treatment. Data from randomized controlled trials comparing stress MPI and CCTA as initial tests in this patient population are only recently becoming available,^7^,^8^ and current practice guidelines do not strongly prefer one modality of testing over the other.^9^,^10^

We performed an international, multi-centric, randomized controlled trial to evaluate the effect of initial testing with stress MPI or CCTA on the use of further downstream testing in patients with suspected CAD. We also compared the costs of the two strategies and the effective radiation dose to patients. Our primary hypothesis was that the initial use of stress-rest MPI would result in less additional noninvasive and invasive testing in the short term.

## Methods

### Study design

This was an open-label, parallel-arm, multi-center, randomized trial, conducted at 6 tertiary care hospitals in 6 countries (Brazil, Czech Republic, India, Mexico, Slovenia, and Turkey) chosen on the basis of expertise in both nuclear imaging and radiology. Randomization was stratified by site and participants’ symptom status (asymptomatic or symptomatic). The study protocol was approved by the ethics committees at all participating sites and all patients provided written informed consent. The study was funded by the International Atomic Energy Agency through a Coordinated Research Project (IAEA-CRP E.1.30.38). The funding agency provided logistic support during the design and conduct of the study but was not involved in the data analysis, interpretation, or the decision to publish. The manuscript was drafted by the lead author with inputs from all investigators and technical experts from the IAEA.

### Participants

Consenting patients above 21 years, who were mildly symptomatic (those in class II NYHA) and had an intermediate likelihood of having CAD,^11^ or asymptomatic patients who were determined to be at intermediate or high risk of coronary events by the Framingham (ATP III) criteria, were eligible to participate. Patients were recruited by treating cardiologists at the outpatient clinics of the participating hospitals. We excluded patients with known CAD, documented either by invasive or non-invasive imaging, a history of myocardial infarction (MI) or coronary revascularization. We also excluded patients who were severely symptomatic (class III or IV NYHA), had chronic renal impairment precluding contrast injection, severe medical disease with limited life-expectancy, known contraindication or allergy to pharmacologic stress agents or contrast agents, or had an abnormal cardiac rhythm (including persistent atrial fibrillation) which precluded ECG gating. Very obese patients were excluded because of weight limitations imposed by scanner design. We did not include pregnant or lactating women.

### Randomization

A random sequence of blocks of varying sizes (4 and 6) stratified by site and symptom status (symptomatic or asymptomatic) was generated using a freely available online random sequence generator (www.randomization.com) by the study statistician at the data management and statistical unit (DMSU), Indian Institute of Public Health-Delhi, India. Allocation was concealed using sequentially numbered, sealed opaque envelopes. Envelopes were prepared by the statistician and sent by post to the recruiting sites. The envelopes contained randomization forms that were completed by the investigator and emailed to the DMSU within 24 hours of randomization. As part of the effort to minimize bias, baseline data including physician preference for either test were recorded after consent was obtained, prior to randomization. Once the allocated diagnostic procedure was known, the patient and referring physician were informed and the procedure was scheduled in consultation with the radiologist or nuclear physician.

### Diagnostic imaging

Stress-rest MPI and CCTA were performed and interpreted by expert nuclear physicians, cardiologists, or radiologists on site. Choice of exercise protocol or pharmacologic stressor agent was left to physician discretion. Images were processed using standard commercially available software. Stress MPI studies were categorized as normal, abnormal, or inconclusive by the reporting nuclear physician. The presence of any perfusion defect (either at rest or stress) or wall motion abnormality (not explained by left bundle branch block) was considered abnormal. In addition, perfusion data were recorded using a 17-segment model and perfusion abnormalities were quantitated using summed scores. Physicians adhered to standard procedures and guideline recommendations while performing stress testing, image acquisition, interpretation, and reporting.^12^–^14^

Coronary CTA studies were performed using a multidetector scanner (64-slice or greater), and reported in accordance with current practice guidelines.^15^,^16^ Calcium scoring was performed prior to contrast injection. Studies were reported as being normal, if there were no coronary stenoses or any luminal narrowing was less than 30% of the reference vessel diameter. Stenoses were categorized as being mild (30%-49%), moderate (50%-69%), or severe (≥70%).

### Data management

All data were entered at participating sites into editable PDF forms with built-in quality checks. The forms were transmitted electronically to the data management center at the Indian Institute of Public Health-Delhi, where the data were exported into statistical analysis software using a customized form management system.

### Study outcome measures

The primary outcome was the proportion of patients having additional non-invasive testing with another modality (rest-stress MPI, CCTA, stress ECG, CMR, or stress ECHO), or invasive coronary angiography within 6 months of initial testing.

Secondary outcomes were as follows: (1) Proportion of patients who had planned, elective invasive angiography at 6-month follow-up; (2) proportion of patients who had planned, elective coronary interventions or bypass surgery at 1-year follow-up; (3) the occurrence of a composite of all-cause mortality, nonfatal MI, recurrent ischemia, or unplanned coronary revascularization at 1-year follow-up; (4) cumulative effective radiation dose (ERD) to patients at 12 months; and (5) total cost of the two strategies at 12 months.

To minimize bias, investigators were explicitly discouraged from performing additional testing with another modality merely to comply with physician preference or local practices. The following were considered acceptable indications for additional non-invasive testing with another modality: (i) Negative initial test, but high clinical suspicion of CAD; (ii) inconclusive initial test result; and (iii) positive initial test, but low clinical suspicion (suspected false positive). As the preference of the referring cardiologist for either of the tests may be an important determinant of further downstream testing, we also adjusted for this variable in the primary analysis. Invasive coronary angiography could be performed in the event of a (i) Positive test (for delineation of anatomy and planning revascularization), (ii) negative initial test but high clinical suspicion, (iii) inconclusive initial test result, (iv) positive initial test but low clinical suspicion (suspected false positive, to rule out CAD).

### Statistical analysis

We hypothesized that either of the strategies would result in 20% of patients receiving a second non-invasive test or coronary angiography during the first 6 months after enrolment. We assumed that we would be able to identify 10-15 sites contributing about 30-40 participants each. We determined that with 500 patients, we would be able to detect a 15% absolute increase in the proportion of patients having a second non-invasive test or coronary angiography between the two strategies, with over 90% power at an alpha level of 0.05, after accounting for a 10% rate of post-randomization loss to follow-up (see Supplementary Material Table 4). However, 7 of the planned 13 sites were not granted ethics or regulatory approval. Further, recruitment rates were lower than expected at most participating sites. The study was stopped due to lack of funding at the end of 3 years after enrolment of 303 patients, without knowledge of the study outcomes. Given that we had only 2% loss to follow-up, this sample size retains 83% power to detect the anticipated difference in the primary outcome between the study groups.

Descriptive statistics are presented for all variables collected at baseline. The primary analysis was by intention-to-treat. In this analysis, all patients whose outcome data were available were included in the arm to which they were randomized, irrespective of the diagnostic procedure received. A per-protocol analysis for the primary outcomes was also performed excluding those who did not undergo the allocated diagnostic procedure, or underwent the procedure 180 days after randomization. The primary outcome was analyzed using logistic regression adjusted for the stratifying factors (site and symptom status) and the stated preference of the treating cardiologist. Odds ratios (OR) and their 95% confidence intervals were computed. Similar analyses were performed for the secondary outcomes. Radiation exposure to each patient undergoing MPI (ERD, effective radiation dose in mSv) was calculated based on the radiopharmaceutical administered and their activities (MBq), as per the most recent recommendations of the International Commission on Radiological Protection.^17^,^18^ For patients undergoing CCTA, ERD was calculated as a product of the dose length product and an organ weighting factor for the chest in accordance with the current recommendations.^17^ For coronary angiography and angioplasty, average ERD values were obtained from the published literature. We used the DRG data from the Slovenian public health system to estimate unit costs for all procedures. Total cost was estimated by addition of direct and indirect costs (data obtained from 49 patients undergoing diagnostic testing at the University Medical Centre, Ljubljana). A *P* value of 0.05 was considered significant. All analyses were performed using Stata 13 (StataCorp LP, College Station, TX, USA).

## Results

### Study Population

**Figure 1:**
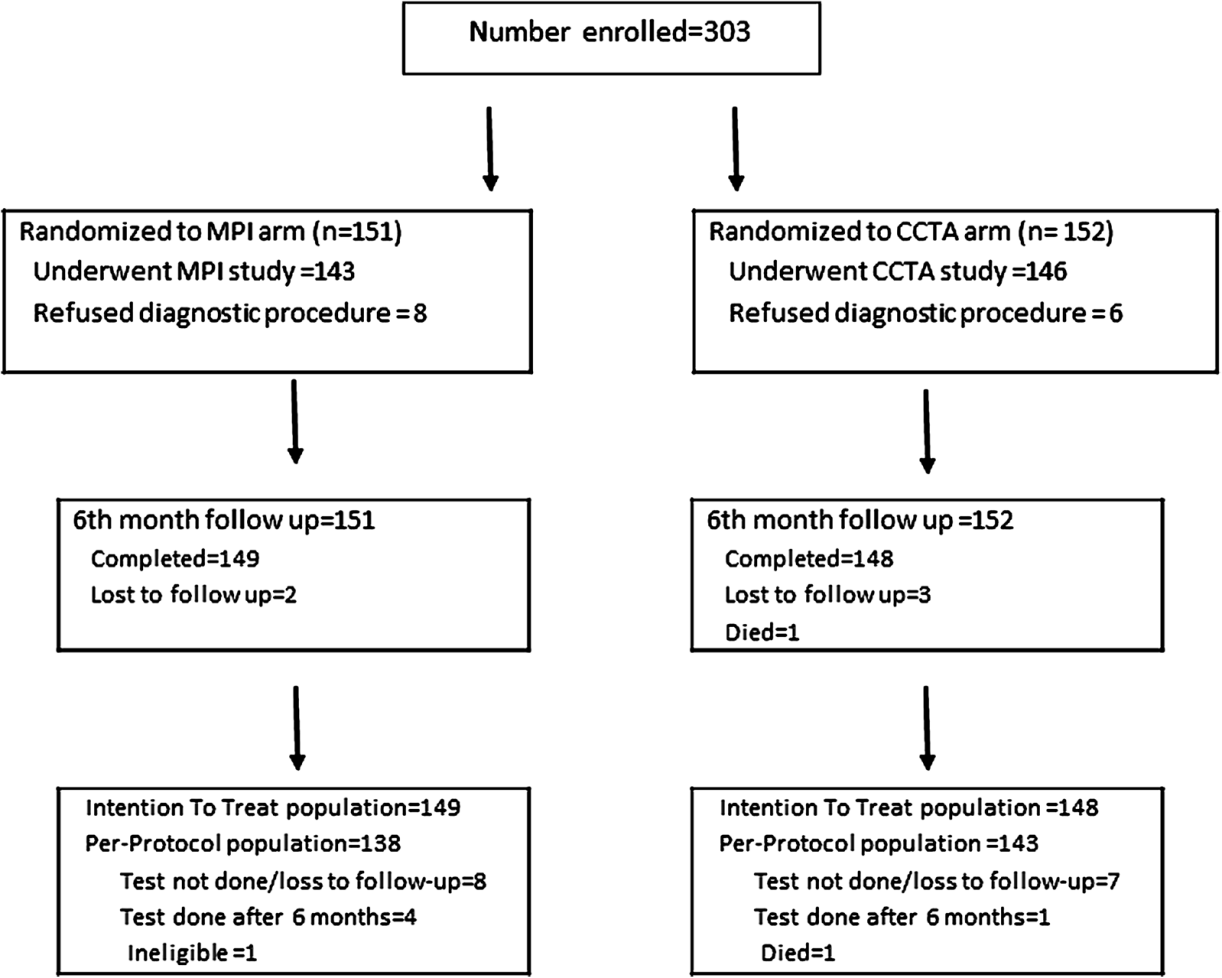
Enrolment, randomization, and follow-up of trial participants. *MPI* Myocardial perfusion imaging, *CCTA* coronary CT angiography

Between June 2011 and 2014, we randomized 303 patients (271, 89.4% symptomatic) at 6 tertiary care hospitals in 6 countries. (Figure 1, Supplementary Material Table 1) The baseline characteristics of included patients were similar in both arms (Table 1). On the average, patients were about 60 years of age, were predominantly male, were overweight, and had a high burden of risk factors for CAD. Notably, nearly 30% of the patients were diabetic, a similar proportion had a family history of premature CAD, nearly 2/3rd were hypertensive, and over half had dyslipidemia. Of the symptomatic patients, chest pain (typical, atypical, or non-anginal) was the commonest symptom (243/271, 90%). Most cardiologists did not have a strong preference for one initial test over the other (Table 1).

**Table 1:** Baseline characteristics

Characteristics	MPI arm (*n* = 151)	CCTA arm (*n* = 152)	*P* value
Age in years	60.2 (11.7)	58.9 (11.1)	0.26
Males	70 (46.4)	75 (49.3)	0.60
Ethnicity			
Caucasian	104 (68.9)	111 (73.0)	
Hispanic	37 (24.5)	31 (20.4)	
Indian	8 (5.3)	8 (5.3)	0.72
African	2 (1.3)	1 (0.7)	
Other	0 (0.00)	1 (0.7)	
BMI	29.0 (9.8)	27.6 (4.4)	0.11
Diabetes	43 (28.5)	43 (28.3)	0.97
Hypertension	97 (64.2)	97 (63.8)	0.94
Smoking	25 (16.6)	36 (23.7)	0.12
Family history of CAD	45 (29.8)	48 (31.6)	0.74
Dyslipidemia	83 (55.0)	89 (58.6)	0.53
Aspirin	76 (50.3)	72 (47.4)	0.61
Statins	76 (50.3)	72 (47.4)	0.61
Beta blockers	62 (41.1)	69 (45.4)	0.45
ACE inhibitors/ARBs	80 (53.0)	90 (59.2)	0.28
Nitrates	21 (16.0)	14 (10.7)	0.20
Diuretics	37 (24.5)	37 (24.3)	0.97
Clopidogrel	6 (4.0)	7 (4.6)	0.79
Calcium channel blocker	26 (17.2)	22 (14.5)	0.51
Antiarrhythmic agents	16 (10.6)	15 (9.9)	0.83
Symptomatic	134 (88.7)	137 (90.1)	0.69
Angina	121	122	
Dyspnea or other ischemic symptoms	13	15	
Non-invasive test preferred by treating physician			
Stress MPI	34 (22.5)	27 (17.8)	
CCTA	17 (11.3)	15 (9.9)	
No preference	100 (66.2)	110 (72.4)	0.50

All continuous variables are reported as mean (standard deviation) and categorical variables as frequency (%)

*MPI* Myocardial perfusion imaging; *CCTA* Coronary computed tomographic angiography; *BMI* body mass index, *CAD* coronary artery disease; *ACE* angiotensin converting enzyme; *ARB* angiotensin receptor blocker

### Initial testing

**Figure 2:**
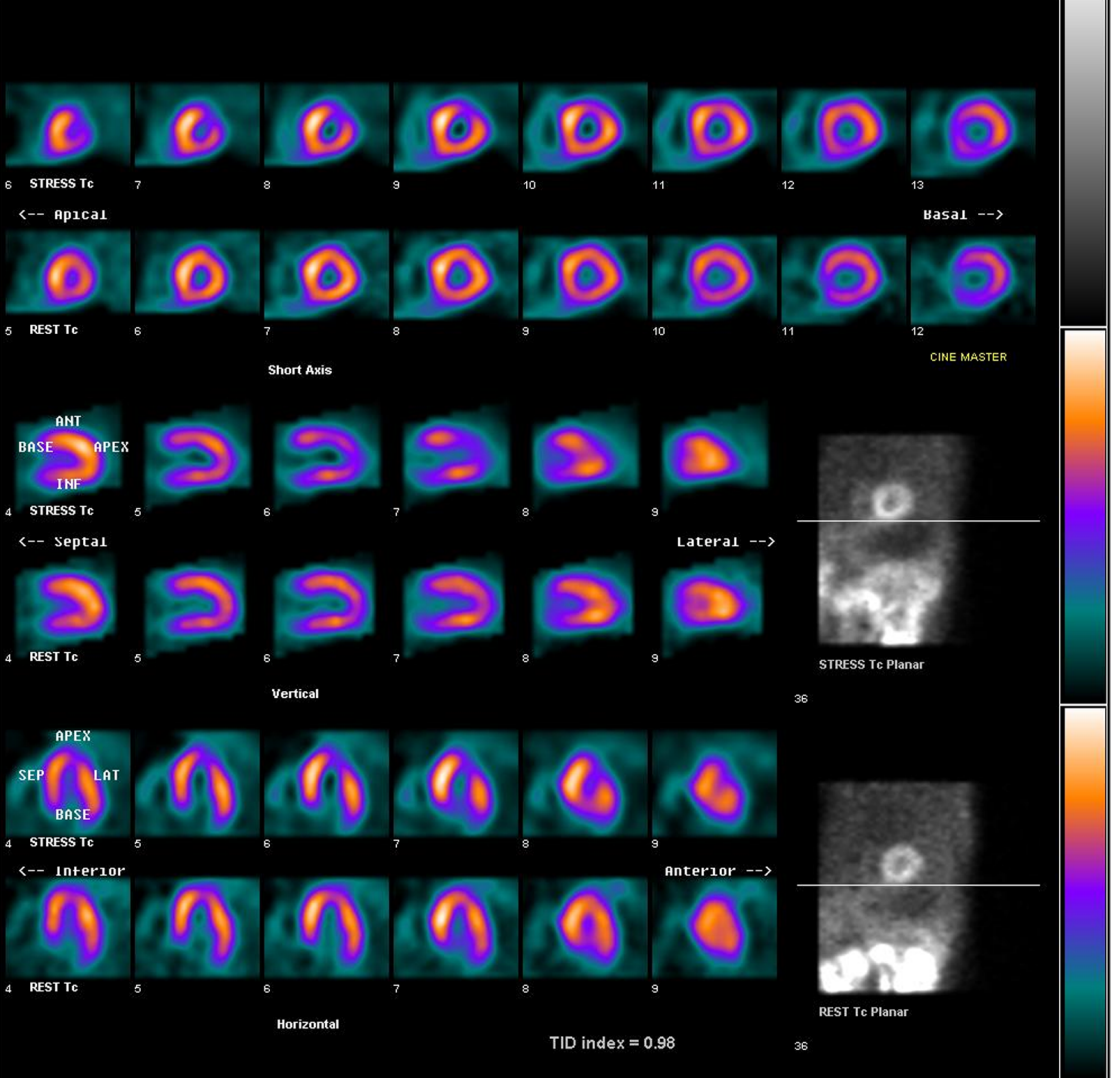
A 73-year-old male with exertional dyspnea and a positive family history of coronary artery disease was randomized to undergo exercise MPI. Stress (*top*) and rest (*bottom*) Tc-99 m tetrofosmin myocardial perfusion images demonstrate reversible ischemia in the apex, apical segment of the anterior wall, and apical segment of the lateral wall. *MPI* myocardial perfusion imaging

**Figure 3:**
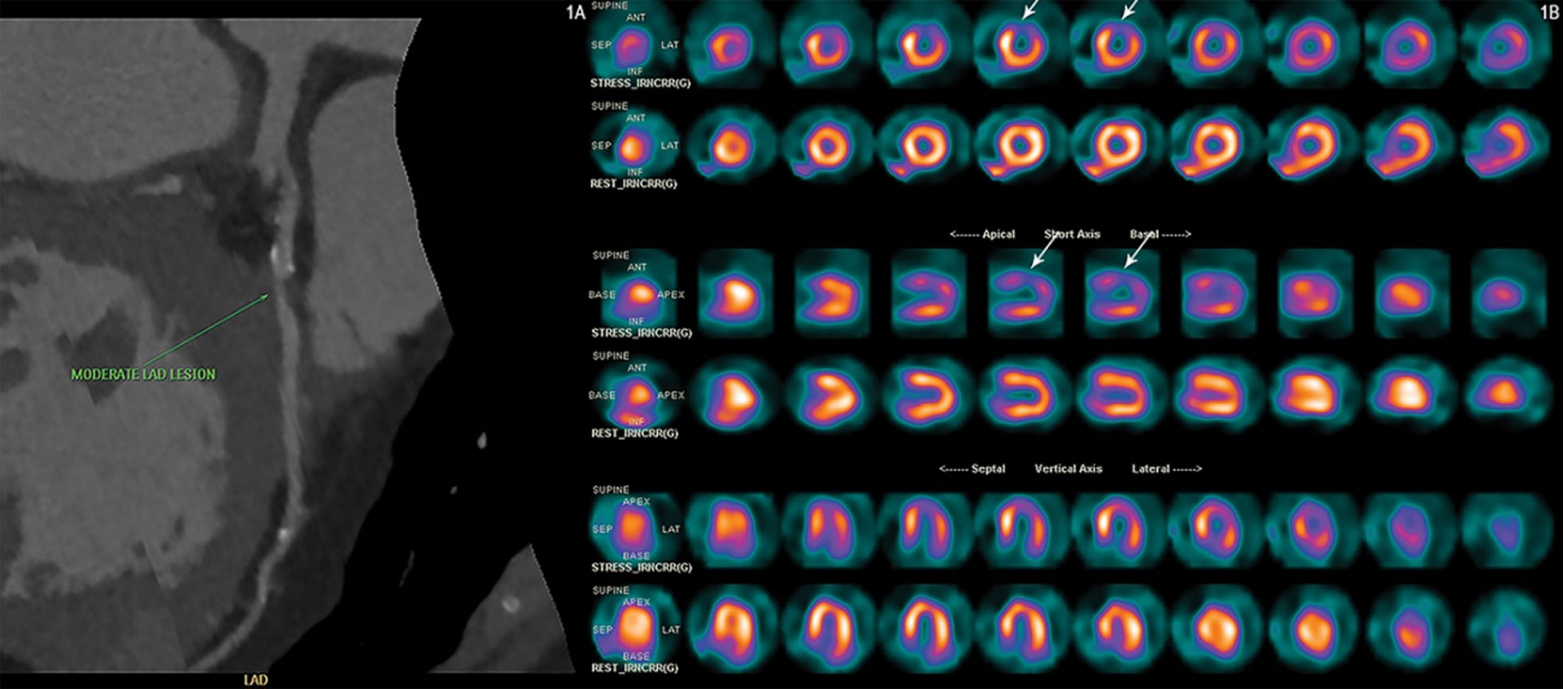
A 68-year-old diabetic male patient with atypical symptoms underwent CCTA which showed a calcium score of 640 Agatston and a partially calcified proximal LAD plaque, causing moderate stenosis (1A -LAD curved multiplanar reconstruction). Subsequent exercise stress MPI revealed severe ischemia (*arrows*) in the anterior wall, antero lateral region and apex (1B). *MPI* myocardial perfusion imaging, *CCTA* coronary CT angiography, *LAD* left anterior descending coronary artery

Hundred and fifty-one patients were randomized to the MPI arm and 152 to the CCTA arm, and 95% underwent testing as allocated (289/303). Details of the study procedures are provided in the Supplementary Material (Representative images in Figures 2 and 3). Most patients had normal initial test results. Forty-one of 143 (29%) patients had an abnormal stress MPI and one patient had an inconclusive result. Of those with abnormal MPI results, 14 (10%) had reversible perfusion defects involving >10% of the LV myocardium and the remaining had less severe defects. An abnormal initial CCTA was reported in 79/141 (56%) patients. Of these, 25 (18%) had at least one lesion with ≥70% diameter stenosis, and 21 (18%) had intermediate lesions (50%-69% diameter stenosis). The median calcium score was 6.7 units with 75% of the patients having a score <97 (Supplementary Material Tables 2, 3).

### Outcomes

Follow-up of at least 6 months was available for 297 (98%) patients. Two patients in the MPI arm and 3 in the CCTA arms were lost to follow-up, and 1 died. One patient was excluded from the analysis as an MPI study had been performed prior to randomization. (Figure 1) Patients undergoing stress MPI as the initial test were half as likely (adjusted OR 0.51, 95% CI 0.28-0.91, *P* = 0.023) as those undergoing CCTA to have the primary outcome. The per-protocol analysis showed similar results (Table 2). In exploratory analyses, the results were consistent (interaction *P* values in parentheses) across subgroups defined by symptom status (*P* = 0.6), angina as presenting symptom (*P* = 0.71), presence of diabetes (*P* = 0.18), and participating site (*P* = 0.10). The difference in the primary outcome was driven largely by the performance of further non-invasive testing to determine the significance of lesions detected on CCTA (Tables 2, 3). Of the 26 patients who underwent repeat non-invasive testing in the CCTA arm, 23 underwent stress MPI. One patient each underwent stress echo, exercise ECG, and cardiac MRI. In the MPI arm, 5 patients underwent a CCTA and 2 underwent repeat MPI after pharmacologic stress. Downstream performance of invasive coronary angiography was not different between the two arms. Most coronary angiograms were done in patients with a positive initial test for the purpose of delineating anatomy and planning revascularization (15/18 in the MPI arm and 20/21 in the CCTA arm).

**Table 2:** Study outcomes

	MPI arm	CCTA arm	OR (95% CI) *P* value	Adjusted OR* (95% CI) *P* value	Adjusted OR^†^ (95% CI) *P* value
Primary outcome (intention-to-treat analysis)^‡^	(*n* = 149)	(*n* = 148)			
	25 (16.8)	41 (27.70)	0.53 (0.30–0.92) 0.025	0.50 (0.28 – 0.89) 0.019	0.51 (0.28 – 0.91) 0.023
Non-invasive testing	7 (4.7)	26 (17.6)	0.32 (0.10-0.55) 0.001	0.19 (0.08-0.48) <0.001	0.18 (0.07-0.46) <0.001
Elective coronary angiography	18 (12.1)	21 (14.2)	0.83 (0.42-1.60) 0.59	0.83 (0.42-1.60) 0.60	0.86 (0.44-1.71) 0.67
Primary outcome (per-protocol analysis)^§^	(*n* = 138)	(*n* = 143)			
	25 (18.1)	41(28.7)	0.55 (0.31, 0.96) 0.038	0.55 (0.31, 0.99) 0.045	0.55 (0.30, 0.98) 0.046
Planned coronary revascularization (CABG/PCI) at 12 months (Intention-to-treat analysis)	(*n* = 129)	(*n* = 124)			–
	10 (7.8)	8 (6.5)	1.22 (0.46, 3.20) 0.69	1.29 (0.48, 3.42) 0.61^†^	

*MPI* Myocardial perfusion imaging; *CCTA* coronary computed tomographic angiography; *CABG* coronary artery bypass grafting; *PCI* percutaneous coronary intervention

* Adjusted for recruiting centers and symptom status

^†^ Adjusted for recruiting centers, symptom status, and physician preference of procedure at baseline

^‡^ Includes all patients whose outcome data were available, analyzed by the group to which they were initially randomized

^§^ Excludes those who did not undergo the allocated diagnostic procedure, or underwent the procedure 180 days after randomization

**Table 3:** Reasons for further non-invasive testing

	MPI arm (*n* = 149)	CCTA arm (*n* = 148)
Non-invasive testing at 6 months	7 (4.7)	26 (17.6)
Negative initial test, but high clinical suspicion	2^*^	4^†^
Inconclusive initial test result	2	21^‡^
Positive initial test, but low clinical suspicion (suspected false positive)	3	1

* One patient had a dilated left ventricle and another had an equivocal perfusion defect (both studies were reported as normal)

^†^ One patient had multiple mild lesions and the others were suspected to have microvascular disease causing angina. All 4 patients had normal subsequent non-invasive test results. No patient had coronary angiography

^‡^ This group includes 10 patients with severe (≥70% diameter stenosis), 5 with intermediate (50%-69% stenosis), and 2 with mild (30%-49%) lesions where the clinician was uncertain about the relationship of the lesions to symptom status. This group also includes one patient with a myocardial bridge involving the left anterior descending artery and 3 patients who could not complete the procedure (2 because of very high calcium scores and 1 because of an allergic reaction to contrast agent)

There were no significant differences in the proportion of patients undergoing coronary angiography at 6 months or any revascularization procedure at 12 months (Table 2). One patient in the CCTA arm died during the follow-up. Two patients in the MPI arm and one in the CCTA arm had recurrent ischemia. One additional patient in the MPI arm underwent unplanned PCI. Overall, the composite of death, nonfatal MI, recurrent ischemia, or unplanned revascularization occurred in 3 (2.3%) patients in the MPI arm and 2 (1.6%) in the CCTA arm.

The median ERD to patients was significantly greater with the initial stress MPI than CCTA (a difference of over 4 mSv). But this difference reduced substantially at 12 months because of more downstream testing in the CCTA arm (Table 4).

**Table 4:** Effective radiation dose to patients

Effective radiation dose (ERD) in mSv	MPI arm	CCTA arm	*P* value
Initial diagnostic procedure	(*n* = 143)	(*n* = 142)	
Median ERD (IQR)	9.3 (8.5, 9.7)	5.0 (3.8, 10)	<0.001^‡^
All diagnostic procedures at 12 months	(*n* = 143)	(*n* = 145)	
Median ERD (IQR)	9.6 (8.9, 12.5)	8.8 (4, 13.2)	0.040^‡^
All diagnostic and therapeutic procedures^*^ at 12 months^†^	(*n* = 143)	(*n* = 145)	
Median ERD (IQR)	9.6 (8.9, 12.5)	8.8 (4, 13.2)	0.041^‡^

* Patients who underwent angiography and percutaneous coronary angioplasty (PCI) at the same time, the dose of PCI was used for calculating the ERD

^†^ For 24 patients in the CCTA and 16 in the MPI arm, 12- month data were unavailable. ERD was estimated from 6-month data for these patients

^‡^ *P* value reported is for the Wilcoxon rank-sum test for the difference in median ERD values

The cost of the initial CCTA was marginally greater than stress MPI (€ 719 vs. 699). However, at 12 months, the average cost per patient was not significantly different between the two arms (€ 1365 vs. 1243, *P* = 0.54) (Supplementary Material Table 5).

## Discussion

The results of this diagnostic randomized trial suggest that in patients with an intermediate likelihood of having CAD, and in those at intermediate or high risk of coronary events, initial evaluation with stress MPI results in substantially less downstream non-invasive and invasive testing before decisions regarding management can be made. There was no difference in costs between the two approaches, but patients evaluated with initial stress-rest MPI received a small but significantly greater cumulative exposure to radiation at 1-year. These results are based on a multi-ethnic population of patients, with a large proportion drawn from emerging economies.

We believe that the validity of these results is enhanced by two important considerations. First, decision-making regarding downstream test use was standardized and pre-specified, and effect-estimates were adjusted for physician preference (for either of the diagnostic modalities), thereby minimizing bias in this diagnostic randomized trial. Second, investigators used contemporary diagnostic equipment and adhered to currently recommended guidelines while performing and reporting test results, thereby reflecting current best practice.

### Increased downstream testing with CCTA

The results of our study are consistent with those from the previous observational and randomized studies. In a systematic review, Nielsen et al^3^ identified 6 observational studies and one small randomized trial^7^ which compared initial functional and anatomical evaluation and reported on downstream test utilization. Combining these results in a meta-analysis, these authors showed that a strategy of initial coronary CTA resulted in greater use of further downstream testing and coronary angiography compared to initial testing with either MPI or exercise ECG (24.4% vs. 18.5%; OR 1.38, 95% CI 1.33-1.43; *P* = 0.0001). The tendency for patients evaluated initially by CCTA to increase the likelihood of downstream coronary angiography (and revascularization) has also been observed in the context of low-risk patients with acute chest pain.^19^,^20^ However, a more recent randomized trial comparing exercise ECG with CCTA in patients with stable chest pain showed a greater use of downstream non-invasive testing in the exercise ECG arm.^21^ This was attributable to the large number of inconclusive exercise ECG results (66/245, 27%). The diagnostic performance of exercise ECG is inferior to stress MPI^22^ and is perhaps not the modality of choice in a comparative evaluation between anatomical and functional testing.

### Downstream testing and coronary revascularization

Much of the increase in downstream testing in the CCTA arm in our study was because of physician uncertainty regarding the relationship between the anatomic lesions seen on CCTA and patient symptoms. The difference in rates of downstream testing was driven primarily by the performance of additional non-invasive tests; there was no difference in the rates of coronary angiography unlike in the previous studies.^3^,^8^,^23^ This may reflect local practices such as a preference for obtaining information from further non-invasive testing rather than from fractional flow reserve (FFR) measurement at angiography.

The increase in rates of coronary angiography also, predictably, increased the rates of revascularization in the CCTA arm in the previous studies. The OR for revascularization with CCTA was 2.6 (2.5-2.77) in the meta-analysis by Nielsen. 3 Likewise, in the Prospective Multicenter Imaging Study for Evaluation of Chest Pain (PROMISE) trial, the rate of revascularization with CCTA was nearly twice that with functional testing (6.2% vs. 3.2%).^8^ We were unable to show any differences in revascularization rates between the two arms perhaps because of a preference for functional testing to decide on significance of lesions, and also partly because of resource constraints limiting the performance of revascularization (in at least 3 of the participating countries, Mexico, India, and Brazil, a large proportion of healthcare spending is out-of-pocket). However the impact of the choice of initial test (and resulting differences in rates of revascularization) on clinical outcomes is unclear. While some studies^3^,^24^ have suggested a reduction in myocardial infarction with a strategy of initial testing with CCTA, the large PROMISE trial and a recent meta-analysis of observational studies of CCTA and MPI did not show any difference in hard clinical outcomes.^8^,^25^

### Effect on radiation exposure and costs

Advances in CT scanner design and improved protocols for image acquisition and analysis have reduced radiation exposure to patients. Expectedly, the median ERD in the CCTA arm was substantially less than that with MPI. However, this difference was attenuated by the greater need for further testing in the CCTA arm by 6 months. But as only a minority of patients in such a cohort are likely to undergo further testing or revascularization by PCI, the distribution of radiation exposure is likely to be complex, and the median ERD may not be a representative measure. Nevertheless, cumulative ERD data from our study are similar to that reported in PROMISE, although the ERD was greater in the CCTA arm in that study.^8^

There were no significant differences in costs between the two strategies. Two previous studies reporting on comparative costs found initial evaluation with CCTA to be cost saving,^26^,^27^ but the initial functional test in these analyses was exercise ECG with its inherently high rate of inconclusive results mandating further testing for decision making.

### Limitations

Our study has several limitations. First, it was prematurely stopped with only 60% of the planned sample recruited and the large effect size seen may therefore reflect a “random-high.” However, our estimates remained stable on adjustment and are likely to indicate a true effect. Second, even though we made efforts to minimize bias both at the design and analysis stage, it is impossible to rule out its effect on physician-driven outcomes in an open-label study. Third, we did not use a central core lab and relied on site-reported test results. Fourth, our study was not powered to detect differences in clinical outcomes which could potentially result from the differences in the rates of downstream testing. Finally, we did not capture information relating to changes in symptom status or medical therapy during the course of follow-up, which may have provided additional insights into the utility of either of the two strategies.

## Conclusions

In the initial evaluation of patients with suspected CAD, a strategy of functional testing with stress-rest MPI compared to CCTA, may result in less downstream testing, but with a small increase in radiation exposure to patients. These results must be taken into consideration when choosing the initial test for the evaluation of patients with suspected CAD.

## New Knowledge Gained

In patients with suspected CAD, initial testing with coronary CTA compared to stress-rest MPI may result in greater downstream test utilization before clinical decisions can be made. Patients being evaluated for suspected CAD should be made aware of the potentially greater requirement for further testing if coronary CTA is used in the initial evaluation

## Electronic supplementary material

Below is the link to the electronic supplementary material.

Supplementary material 1 (DOCX 25 kb)

Supplementary material 2 (PPTX 392 kb)

## Abbreviations

MPI: Myocardial perfusion imaging
CCTA: Coronary computerized tomography angiography
CAD: Coronary artery disease
IAEA: International Atomic Energy Agency
ATP III: Adult Treatment Program III
CMR: Cardiac magnetic resonance
ECG: Electrocardiogram
NYHA: New York Heart Association
ERD: Effective radiation dose
