# Supplementary material for: Functional compared to anatomical imaging in the initial evaluation of patients with suspected coronary artery disease: An international, multi-center, randomized controlled trial (IAEA-SPECT/CTA study)
Source: J Nucl Cardiol. 2016 Oct 28;24(2):507–17. doi: 10.1007/s12350-016-0664-3 (PMC5413523; doi:10.1007/s12350-016-0664-3)
Supplement: Supplementary file 1 — Supplementary material 1 (DOCX 25 kb) [file 12350_2016_664_MOESM1_ESM.docx]

**SUPPLEMENTARY APPENDIX**

**Functional compared to anatomical imaging in the initial evaluation of patients with suspected coronary artery disease: An international, multi-center, randomized controlled trial (IAEA-SPECT/CTA study)**

Karthikeyan et al.

**Table 1: Participating clinical sites, principal investigators and number of study participants**

| **Site, city, country** | **Principal investigator/s** | **Number of patients recruited** |
| --- | --- | --- |
| Quanta Diagnóstico & Terapia, Curitiba, Brazil | J Vitola | 28 |
| Faculty Hospital Ostrava, Ostrava, Czech Republic | O Kraft | 22 |
| All India Institute of Medical Sciences, New Delhi, India | G Karthikeyan | 17 |
| Ignacio Chávez National Institute of Cardiology, Mexico City, Mexico | E Alexanderson | 68 |
| University Medical Centre Ljubljana, Slovenia | B Guzic-Salobir, B Jug | 143 |
| Ankara University Medical Faculty, Ankara, Turkey | E Ozkan | 25 |

**Study definitions**

Non-fatal myocardial infarction was defined as a typical rise and fall in concentrations of creatinine kinase-MB with at least one of the following: ischemic symptoms, development of pathological Q waves, or ischemic electrocardiographic changes. One value of elevated cardiac troponin was considered sufficient evidence of nonfatal MI if accompanied by any of the above symptoms or ECG changes.

Recurrent ischemia was defined as the occurrence of new anginal symptoms or worsening of angina documented by the physician or by a new (i.e. initial test was negative) positive stress test result during the period of follow-up. Increase in severity of perfusion defects or coronary stenoses were not considered as evidence of recurrent ischemia.

Unplanned coronary revascularization was defined as the performance of percutaneous angioplasty or coronary bypass surgery not directly resulting from the result of the initial noninvasive test result.

**Details of initial testing**

**Table 2: Procedural details and results of initial stress MPI**

| **Characteristics** | **(n=151)** |
| --- | --- |
| Diagnostic procedure done as scheduled | 143 (95.3) |
| Rest Parameters  ECG Normal  ECG Abnormal | 107 (74.8)  36 (25.2) |
| Stress type exercise  Exercise type  Treadmill  Bicycle  Protocol type  Bruce  Other  Exercise duration  Peak HR (beats/min)  % of maximal predicted heart rate  Peak exercise systolic BP, mm Hg  Peak exercise diastolic BP, mm Hg    Reason for exercise termination  Exhaustion  Max predicted  Others  Symptoms/ ECG changes  High BP | 87 (60.8)  50 (57.5)  37 (42.5)  58 (66.7)  29 (33.3)  8.29 (3.2)  151 (16)  96.3 (16.3)  176 (34)  89 (12)  48 (55.2)  24 (27.6)  8 (9.2)  6 (6.9)  1 (1.2) |
| Pharmacologic  Type of stress  Dipyridamole  Adenosine  Peak HR (beats/min)  Post stress systolic BP, mm Hg  Post stress diastolic BP, mm Hg  Reason for stress termination  Protocol  ECG changes  Symptoms | 56 (39.2)  51 (91.1)  5 (8.9)  91 (16)  135 (20)  80 (12)  51 (91.1)  1 (1.8)  4 (7.1) |
| Exercise/stress ECG  Normal  Abnormal  Inconclusive | 89 (62.2)  29 (20.3)  25 (17.5) |
| **MPI results** | **(n=143)** |
| Protocol type  Two day  One day rest stress  One day stress- rest  Stress only | 94 (65.7)  25 (17.5)  14 (9.8)  10 (7.0) |
| Radiopharmaceutical agent  Tetrofosmin  MIBI | 79 (55.2)  64 (44.8) |
| Rest activity (n=133) | 543.7 (214.3) |
| Stress activity | 607.5 (204.6) |
| SPECT result (n=143)  Normal  Abnormal  Inconclusive | 101 (70.6)  41 (28.7)  1 (0.7) |
| Total reversible defect % (n=140)  0  1-4  5-9  10 and above | 101 (72.1)  10 (7.1)  15 (10.7)  14 (10) |
| Adverse events | 0 (0) |

All continuous variables reported as mean (1SD) and categorical variables as frequencies (%)

**Table 3: Procedural details and results of initial coronary CTA**

| **CCTA REPORT** | **(n=152)** |
| --- | --- |
| Diagnostic procedure done as scheduled | 146^*^  (96.1) |
| Prospective gating protocol | 91 (64.1) |
| Effective radiation dose | 7.80 (6.5) |
| Premedication used | 77 (54.2) |
| Acquisition heart rate | 66 (10) |
| Mean calcium score (n=130)  Median calcium score (IQR) | 270.6 (478.9)  6.7 (0, 97) |
| Adverse event ^†^ | 3 (2.10) |
| **CTA report** | **n=141** ^‡^ |
| Normal (no stenosis) | 62 (43.9) |
| Mild (30-49) | 33 (23.4) |
| Moderate (50-69) | 21 (14.9) |
| Severe(>=70) | 25 (17.7) |

* 4 had only Ca scores

†Allergic reaction, extravasation

‡ 6 test not done, 4 only calcium scoring, 1 contrast reaction

**Sample size calculation**

**Table 4: Sample size matrix**

| **Difference in primary outcome** | **Sample size for 80% power** | **Sample size for 90% power** |
| --- | --- | --- |
| 10% | 653 | 811 |
| 15% | 306 | 411 |
| 20% | 182 | 242 |

Sample size calculations for both arms together accounting for 10% loss to follow-up (α = 0.05). We assumed that ordinarily, about 20% of patients would undergo subsequent noninvasive testing or coronary angiography

**Cost calculation**

Unit costs for both diagnostic and interventional procedures were obtained from DRGs reported in the Slovenian public health system. Patients undergoing noninvasive diagnostic procedures (22 CCTA and 27 SPECT) at the University Medical Centre, Ljubljana were interviewed to estimate direct and indirect costs to patients. Direct costs included the cost of travel to the medical center and cost of meals. Travel costs were self-reported or obtained from standard public transport charges. For patients who drove to the center, we estimated the cost based on the distance from their homes and also included parking fees. Indirect costs were estimated from lost wages for the patient (and any accompanying persons).

All the SPECT procedures performed at the University Medical Center followed a two-day protocol. No patient stayed overnight and therefore the direct costs for SPECT include the cost of travel for two visits. Patients are hospitalized on average for 3 days for PCI and 7 days for CABG. Average per-day direct and indirect costs for these procedures were estimated from daily costs incurred by patients undergoing noninvasive testing.

**Table 5: Estimated total procedure costs (in Euros)**

|  | **Procedure + Hospitalization cost** | **Hospitalization Days** | **Mean direct patient cost** | **Mean indirect patient cost** | **Total** |
| --- | --- | --- | --- | --- | --- |
| SPECT | 591.48 |  | 39.79 | 67.75 | 699.03 |
| CCTA | 620.86 |  | 24.27 | 73.77 | 718.90 |
| Coronary angiogram | 1510.71 | 1 | 24.27 | 73.77 | 1608.75 |
| PCI without stent | 2670.09 | 3 | 35.91 | 221.31 | 2927.31 |
| PCI with stent | 3302.48 | 3 | 35.91 | 221.31 | 3559.70 |
| PCI, complicated | 7149.52 | 3 | 35.91 | 221.31 | 7406.74 |
| CABG | 13016.7 | 7 | 59.18 | 516.39 | 13592.27 |
| CABG, complicated | 17794.76 | 7 | 59.18 | 516.39 | 18370.33 |

All costs are in Euros. SPECT is myocardial perfusion imaging performed by single photon emission computed tomography; CCTA - Coronary computed tomographic angiography; PCI - Percutaneous coronary angiography; CABG - Coronary artery bypass grafting. Complicated PCI or CABG refers to procedures complicated by the occurrence of “catastrophic comorbidities and/or catastrophic complications, and/or patient is older than 65 years” referenced from the DRGs for Slovenia
