# Supplementary material for: Functional compared to anatomical imaging in the initial evaluation of patients with suspected coronary artery disease: An international, multi-center, randomized controlled trial (IAEA-SPECT/CTA study)
Source: J Nucl Cardiol. 2016 Oct 28;24(2):507–17. doi: 10.1007/s12350-016-0664-3 (PMC5413523; doi:10.1007/s12350-016-0664-3)
Supplement: Supplementary file 2 — Supplementary material 2 (PPTX 392 kb) [file 12350_2016_664_MOESM2_ESM.pptx]

## Slide 1
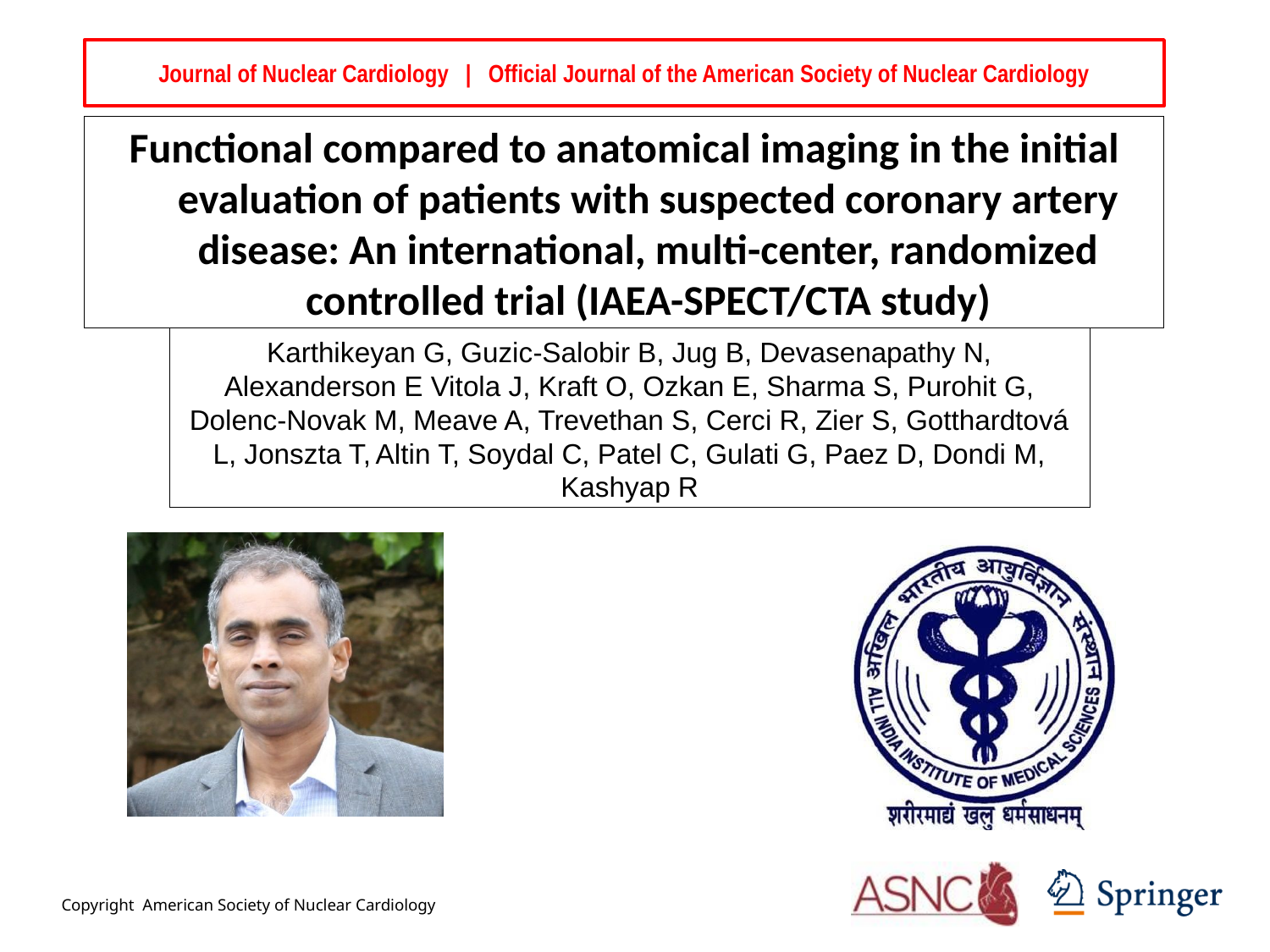

Journal of Nuclear Cardiology | Official Journal of the American Society of Nuclear Cardiology
# Functional compared to anatomical imaging in the initial evaluation of patients with suspected coronary artery disease: An international, multi-center, randomized controlled trial (IAEA-SPECT/CTA study)
Karthikeyan G, Guzic-Salobir B, Jug B, Devasenapathy N, Alexanderson E Vitola J, Kraft O, Ozkan E, Sharma S, Purohit G, Dolenc-Novak M, Meave A, Trevethan S, Cerci R, Zier S, Gotthardtová L, Jonszta T, Altin T, Soydal C, Patel C, Gulati G, Paez D, Dondi M, Kashyap R
Copyright American Society of Nuclear Cardiology

## Slide 2
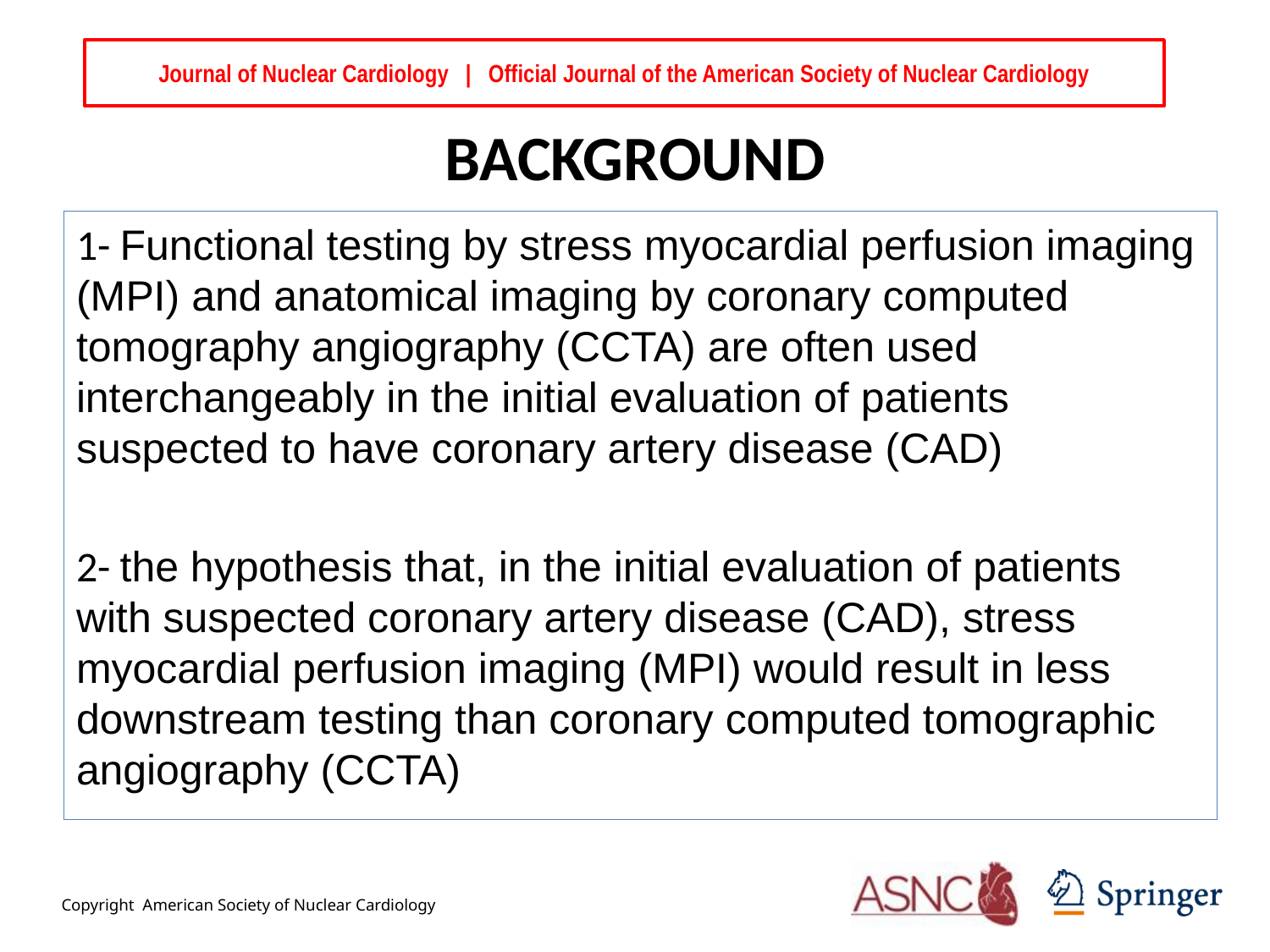

Journal of Nuclear Cardiology | Official Journal of the American Society of Nuclear Cardiology
# BACKGROUND
1- Functional testing by stress myocardial perfusion imaging (MPI) and anatomical imaging by coronary computed tomography angiography (CCTA) are often used interchangeably in the initial evaluation of patients suspected to have coronary artery disease (CAD)
2- the hypothesis that, in the initial evaluation of patients with suspected coronary artery disease (CAD), stress myocardial perfusion imaging (MPI) would result in less downstream testing than coronary computed tomographic angiography (CCTA)
Copyright American Society of Nuclear Cardiology

## Slide 3
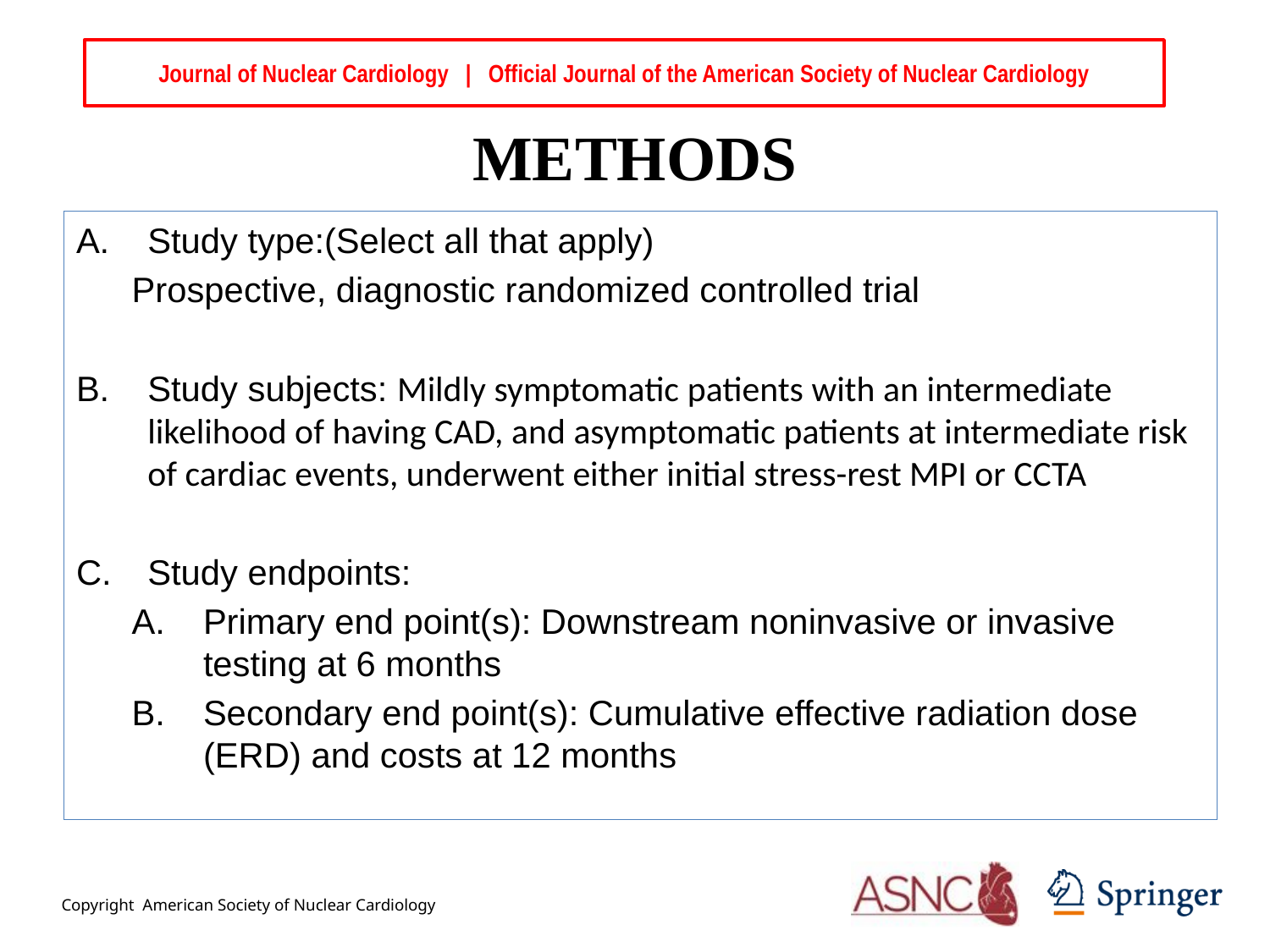

Journal of Nuclear Cardiology | Official Journal of the American Society of Nuclear Cardiology
# METHODS
Study type:(Select all that apply)
Prospective, diagnostic randomized controlled trial
Study subjects: Mildly symptomatic patients with an intermediate likelihood of having CAD, and asymptomatic patients at intermediate risk of cardiac events, underwent either initial stress-rest MPI or CCTA
Study endpoints:
Primary end point(s): Downstream noninvasive or invasive testing at 6 months
Secondary end point(s): Cumulative effective radiation dose (ERD) and costs at 12 months
Copyright American Society of Nuclear Cardiology

## Slide 4
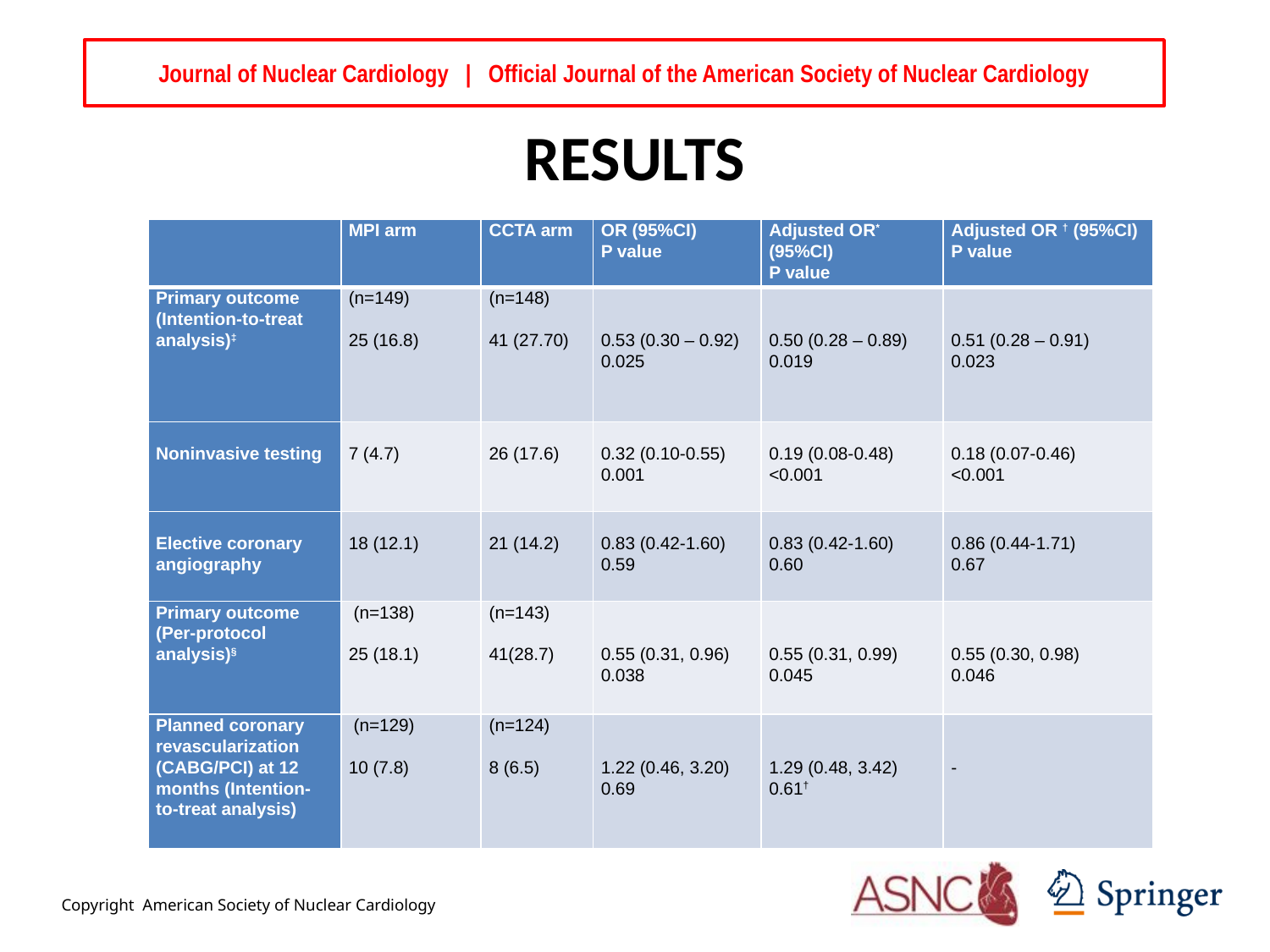

Journal of Nuclear Cardiology | Official Journal of the American Society of Nuclear Cardiology
# RESULTS
| | MPI arm | CCTA arm | OR (95%CI) P value | Adjusted OR\* (95%CI) P value | Adjusted OR † (95%CI) P value |
| --- | --- | --- | --- | --- | --- |
| Primary outcome (Intention-to-treat analysis)‡ | (n=149)   25 (16.8) | (n=148)   41 (27.70) | 0.53 (0.30 – 0.92) 0.025 | 0.50 (0.28 – 0.89) 0.019 | 0.51 (0.28 – 0.91) 0.023 |
| Noninvasive testing | 7 (4.7) | 26 (17.6) | 0.32 (0.10-0.55) 0.001 | 0.19 (0.08-0.48) <0.001 | 0.18 (0.07-0.46) <0.001 |
| Elective coronary angiography | 18 (12.1) | 21 (14.2) | 0.83 (0.42-1.60) 0.59 | 0.83 (0.42-1.60) 0.60 | 0.86 (0.44-1.71) 0.67 |
| Primary outcome (Per-protocol analysis)§ | (n=138)   25 (18.1) | (n=143)   41(28.7) | 0.55 (0.31, 0.96) 0.038 | 0.55 (0.31, 0.99) 0.045 | 0.55 (0.30, 0.98) 0.046 |
| Planned coronary revascularization (CABG/PCI) at 12 months (Intention-to-treat analysis) | (n=129)   10 (7.8) | (n=124)   8 (6.5) | 1.22 (0.46, 3.20) 0.69 | 1.29 (0.48, 3.42) 0.61† | - |
Copyright American Society of Nuclear Cardiology

## Slide 5
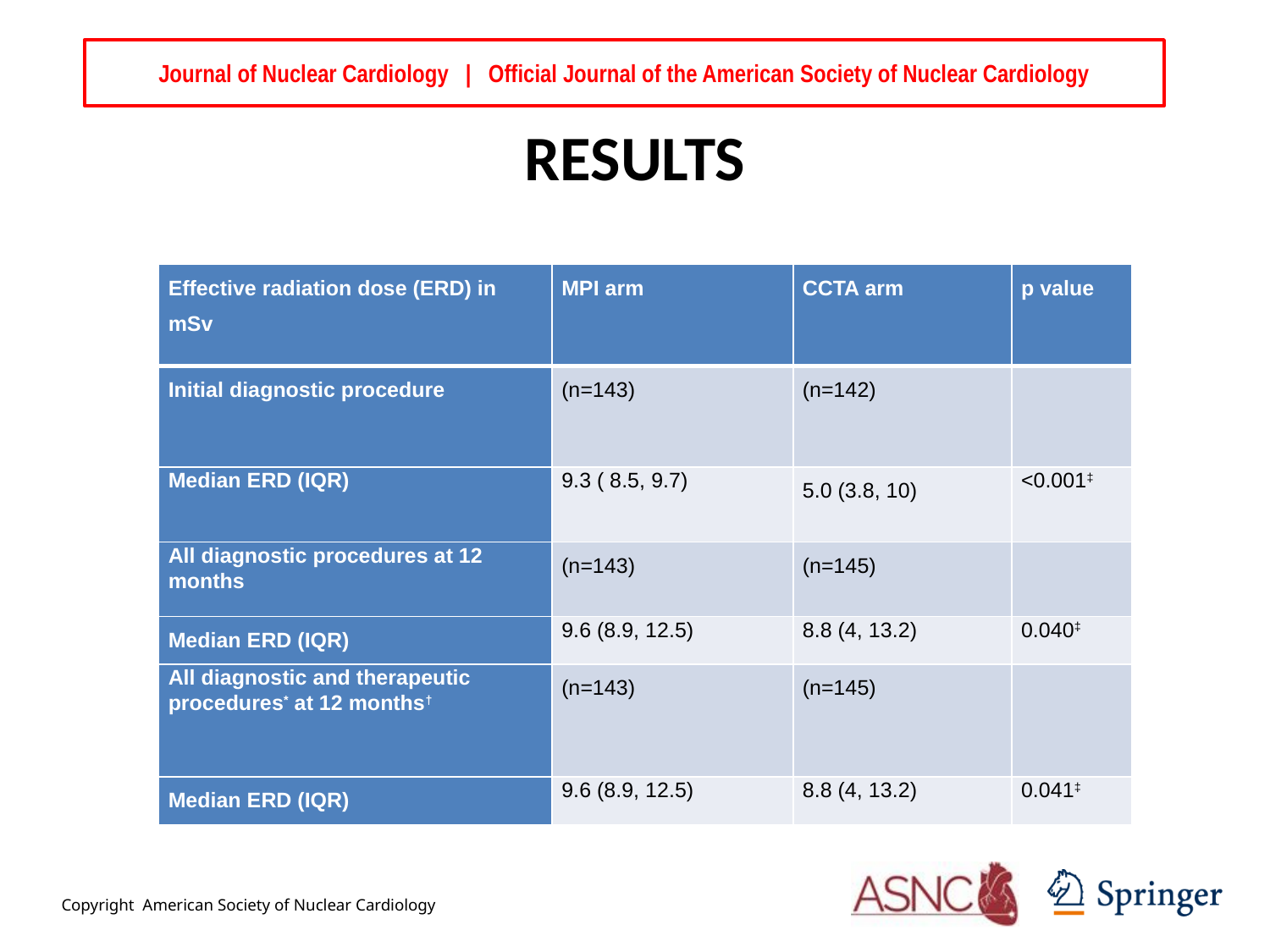

Journal of Nuclear Cardiology | Official Journal of the American Society of Nuclear Cardiology
# RESULTS
| Effective radiation dose (ERD) in mSv | MPI arm | CCTA arm | p value |
| --- | --- | --- | --- |
| Initial diagnostic procedure | (n=143) | (n=142) | |
| Median ERD (IQR) | 9.3 ( 8.5, 9.7) | 5.0 (3.8, 10) | <0.001‡ |
| All diagnostic procedures at 12 months | (n=143) | (n=145) | |
| Median ERD (IQR) | 9.6 (8.9, 12.5) | 8.8 (4, 13.2) | 0.040‡ |
| All diagnostic and therapeutic procedures\* at 12 months† | (n=143) | (n=145) | |
| Median ERD (IQR) | 9.6 (8.9, 12.5) | 8.8 (4, 13.2) | 0.041‡ |
Copyright American Society of Nuclear Cardiology

## Slide 6
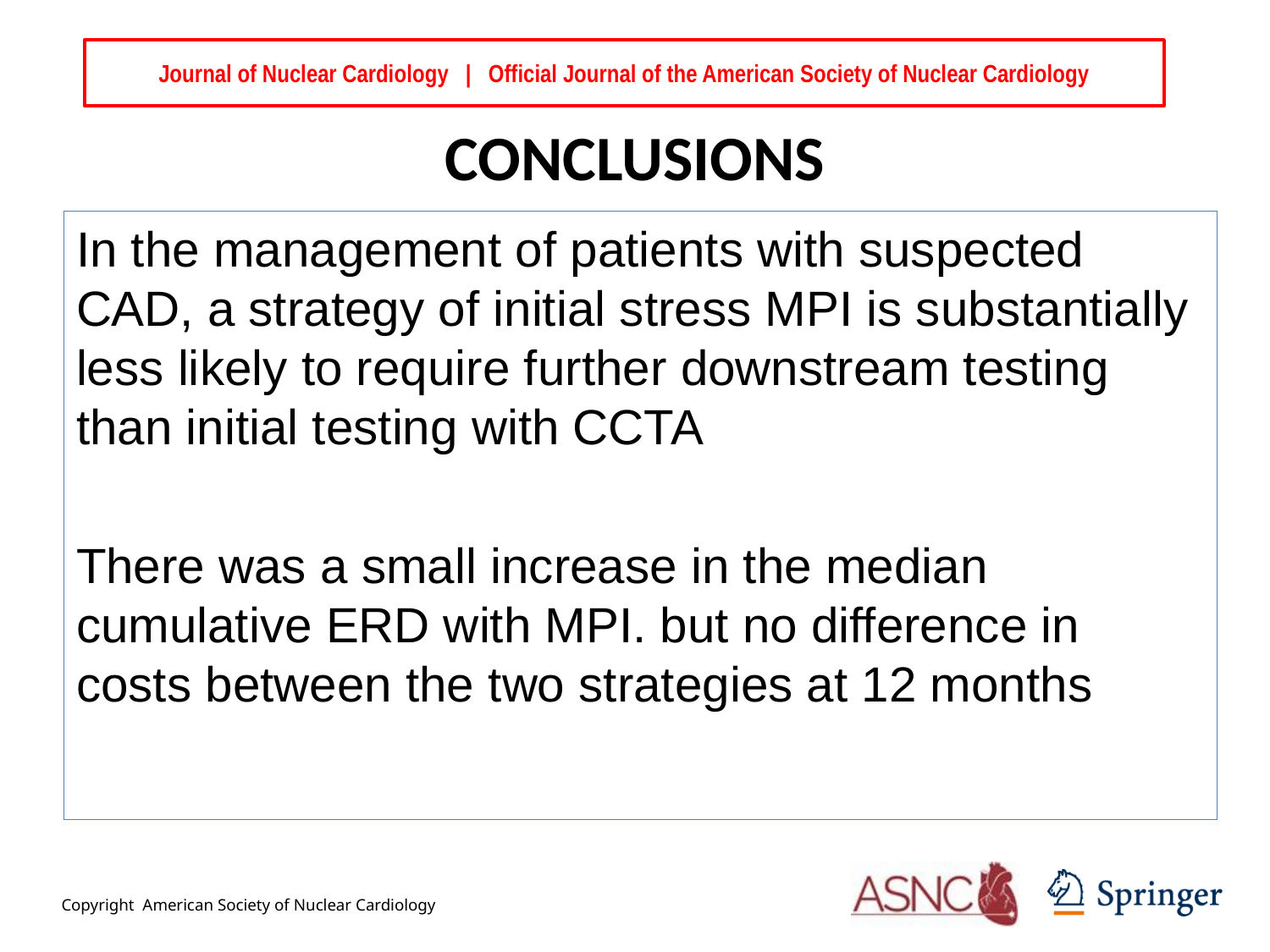

Journal of Nuclear Cardiology | Official Journal of the American Society of Nuclear Cardiology
# CONCLUSIONS
In the management of patients with suspected CAD, a strategy of initial stress MPI is substantially less likely to require further downstream testing than initial testing with CCTA
There was a small increase in the median cumulative ERD with MPI. but no difference in costs between the two strategies at 12 months
Copyright American Society of Nuclear Cardiology
